# Integrating transient cellular and nuclear motions to comprehensively describe cell migration patterns

**DOI:** 10.1038/s41598-018-19885-y

**Published:** 2018-01-24

**Authors:** Tian Lan, Shen-Hsiu Hung, Xudong Su, Samuel W. K. Wong, Yiider Tseng

**Affiliations:** 1 Department of Chemical Engineering, University of Florida, Gainesville, FL 32611 USA; 2 Department of Statistics, University of Florida, Gainesville, FL 32611 USA; 3 J. Crayton Pruitt Family Department of Biomedical Engineering, University of Florida, Gainesville, FL 32611 USA; 4 Institute for Cell & Tissue Science and Engineering, University of Florida, Gainesville, FL 32611 USA; 5 National Cancer Institute-Physical Science Oncology Center, Gainesville, FL 32611 USA

## Abstract

Various subcellular activities, such as protrusion and detachment, compose a cell migration process. The molecular mechanisms of these subcellular activities have been elucidated. However, there is no method that can assess the contributions of these subcellular activities to the global cell migration pattern of a given cell type. Hence, we develop a powerful approach based on *CN correlations* that quantitatively profiles the cell migration pattern of a given cell type in terms of assembled subcellular activities. In this way, we bridge migration data at the cellular level with underlying molecular mechanisms. The *CN correlation* profile is found to uniquely and consistently represent the cell migration pattern of each cell type probed. It can clearly reveal the effects of molecular perturbations, such as Y27632 and Cdc42 knockdown on each subcellular migratory activity. As a result, the *CN correlation* approach serves as a cell dynamic descriptor that can extract comprehensive quantitative data from cell migration movies for integrative biological analyses.

## Introduction

Translational research anticipates a thorough connection of information from cells and beyond to explain life processes and pathological events at the whole organism level^1,2^. Yet, current methods cannot effectively determine the spatiotemporal relationships among various signaling pathways to draw a comprehensive picture of cell physiology. Hence, the elucidation of the relationships for a cluster of proteins becomes an emerging goal for methodological developments^3^. With this evolution, integrated biology has an emphasis on incorporating information from genomics, transcriptomics, proteomics, *etc*. to unravel the complicated crosstalk among signaling pathways and its impacts on the final cellular outcomes. The integrated view of the roles of various proteins in certain subcellular activities has been emphasized in previous studies to understand the migratory subcellular activities^4^. However, more versatile integrated approaches are still needed to effectively streamline subcellular activities for the insight of cellular behaviors on a larger scale^5^.

When considering cell migration, current cellular assessments, including persistence time analysis, directionality, and the wound-healing assay, treat each individual cell as a simple object and either utilize their centroid trajectories or the space occupancy to analyze cell motility^6–11^. These approaches overlook the contributions of the composed subcellular activities, such as actomyosin contraction and lamellipodium formation, on cell motility. Yet, these subcellular activities are the ones that have the underlying molecular mechanisms elucidated and could be used to directly bridge the cell migration status with the signaling pathways behind it. These subcellular activities are mainly governed by the Rho GTPases – RhoA, Rac1, and Cdc42 – which interact with each other through crosstalk and individually have varying degrees of contributions to all subcellular migratory activities^12,13^. To summarize briefly, RhoA plays a dominant role in actomyosin contraction to promote the trailing edge detachment from the substrate for migratory cells^14^. Rac1 is critical for triggering the formation of lamellipodia to promote cell protrusion^15^. Lastly, Cdc42 is mainly responsible for guiding the microtubule-cytoskeleton in the leading edge of cells to maintain cell polarity^16–18^. Hence, if cell migration can be profiled in a way that its underlying subcellular activities can be broken down with high spatiotemporal resolution, our integrated view of cell migration should be greatly enhanced.

A migratory cell alternatively protrudes its leading edge toward new territory and detaches its trailing edge from the anchored substrate^12,19^. During these processes, the nucleus possesses different displacement modes: in protrusion, the nucleus often remains stationary, and in detachment, the nucleus moves along with the main cell body toward the leading edge. Hence, the nuclear centroid displacement (*NCD*) relative to the coupled cell centroid displacement (*CCD*) might uniquely characterize these subcellular migratory activities. In our previous study^20^, we proposed the cell-nucleus (*CN*) *correlation* for calculating a cell migration potential index (*CMPI*) to describe the motility of single cells, and used average *CMPI* values to summarize the motilities of different cell types. Here, we further extend the *CN correlation* approach from the single cell *CMPI* metric to an analysis of cell migration patterns, by pooling together data from single cells to profile different cell types with a statistical modeling approach.

Once the overall cell migration pattern of a cell type is profiled through these coupled motions, the unique signature of the cell migration pattern for individual cell types might be revealed. In this way, a quantitative description for cell migration can be developed. Through combining this development with the results from current molecular approaches, we anticipate progress towards a novel integrated biology approach that includes a quantifiable and comprehensive cell-to-molecular correspondence for analyzing cell migration in different cell conditions.

## Results

### Each exampled subcellular migratory activity has a specific distribution of *CN correlations*

To determine whether the *NCD* relative to the coupled *CCD* can uniquely characterize different subcellular migratory activities, we analyzed all the available subcellular activities identified in the NIH 3T3 fibroblast movies. For each type of subcellular activity, at least 5 sets of movies were analyzed. In these movies, cells and coupled nuclei were labeled using red fluorescent protein (RFP) and Hoechst 33342, respectively, and simultaneously recorded at one-minute time intervals to document appropriate cell dynamics. Consequently, we extracted the momentary cell centroid displacement (*CCD*) and the coupled *NCD* along the *CCD* (*NCD*_//_) for analysis. We focused on the link between *CCD* and the coupled *NCD*_//_ because these two displacements were aligned to the direction of the forces contributing to cell migration.

The coupled pair (*CCD, NCD*_//_) defines what we call a *CN correlation* and can be visualized as a coordinate point on a *CCD vs. NCD*_*//*_ plot (*CN* plot). *CN* plots of *CN correlations* extracted from sequences of a specific subcellular migratory activity might then have a unique distribution profile that can be distinguished from those extracted from other subcellular activities.

**Figure 1:**
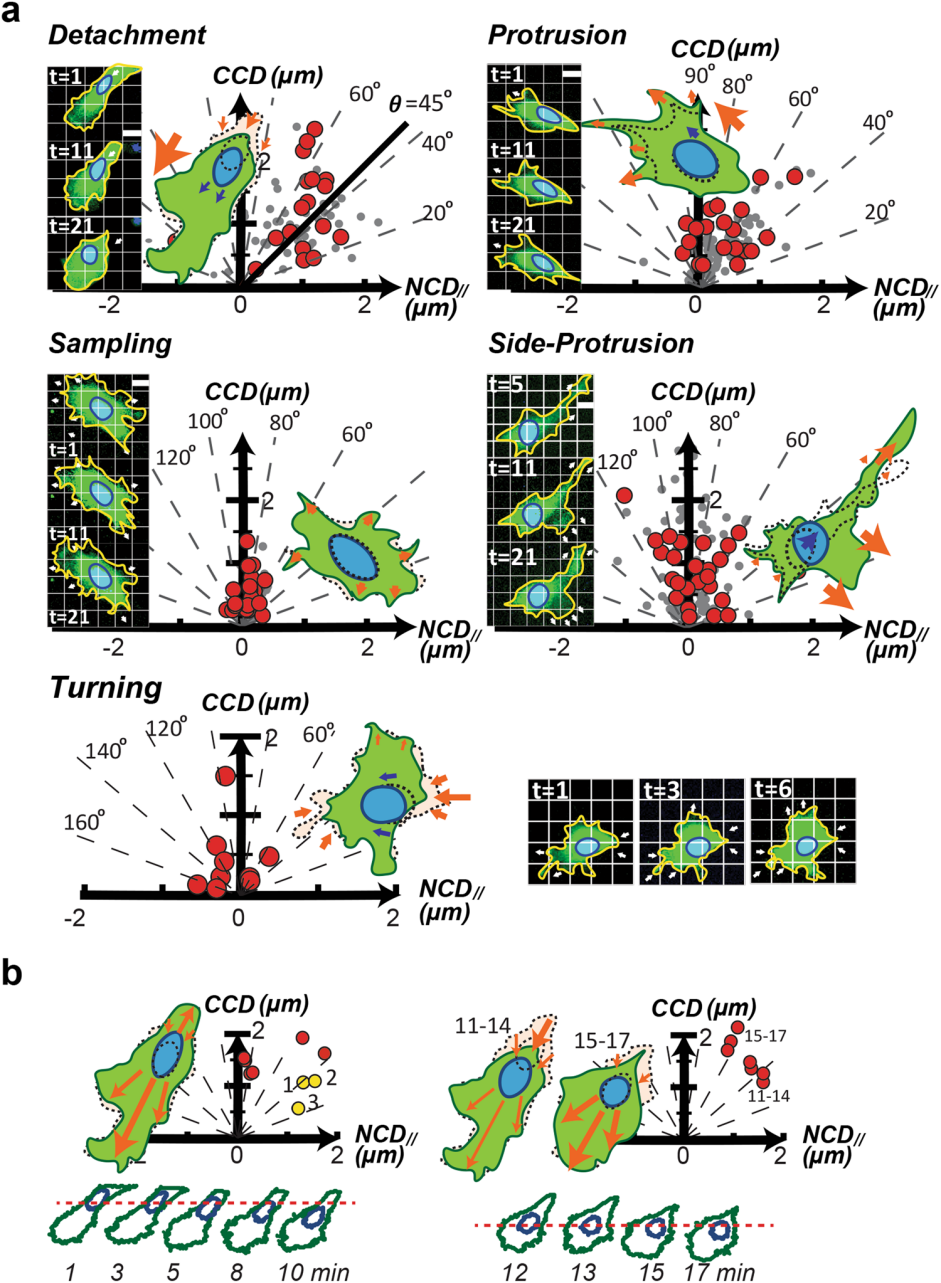
The *CN correlation* data extracted from each of the subcellular migratory activities has a specific distribution in the *CN* plot. (**a**) Stack-images of fluorescently labeled NIH 3T3 cells (green) and coupled nuclei (blue) of each subcellular migratory activity (Supplementary Videos S1–S5), were analyzed, where the images are displayed in a grim graph to depict the cell and nuclear motion (left). The corresponding *CN correlation* distributions are exhibited by red dots in a *CN* plot, where the gray dots are from other events of the same subcellular activity (*right*). (**b**) *Top:* The two panels depict the step-evolution of the detachment event. Yellow dots: the first three data. *Bottom:* The outlines of cell (green) and nucleus (blue) display the peripheral dynamics of the detachment events during the observed time. The red dashed line addresses the same position.

Interestingly, the distributions of these subcellular activities can be distinguished clearly using polar coordinates in the *CN plot*. The trailing edge detachment, leading edge protrusion, pure protrusion (cell sampling), large-angle side protrusion, and cell contraction events each has a specific polar angle zone in the *CN* plot. These zones are mainly between [20°, 70°], [60°, 90°], [60°, 120°], [90°, 130°], and [130°, 170°], respectively. Even though the polar angle distributions of different subcellular activities may have a certain degree of overlap, these distributions concentrate in different distances from the pole (Fig. 1a). In general, *CN correlations* of detachment events have the farthest distance from the pole, followed by those of leading-edge protrusion and side protrusion, and finally those of sampling and contraction events are closest to the pole.

The evolution in the position of *CN correlations* during a subcellular activity is also useful for understanding the mechanical mechanism of that activity. Using a detachment event as an example (Fig. 1b): at the beginning of the event, the *CN correlations* were mainly located between 20°–45° polar angles, which suggested that *NCD*_//_ > *CCD*. Indeed, the movie showed that the detachment-predominant event started from a minor leading edge protrusion while the nucleus also exhibited a steady forward motion. However, the main cell anchorage sites in the trailing edge were steadily attached to the substrate. Later on, the *CN correlations* shifted closer to ~60° polar angles (*CCD* > *NCD*_//_). During the time, the anchorage detached from the substrate, but the *NCD*_//_ values were mainly generated from the relaxation of nucleus (from elongated to rounded) without forward motion. Some other examples are provided in the Supplementary Information.

### The *CN correlation* profile of a cell type generated from sufficient data is consistent and unique

It would not be obvious that cell migration properties of individual cells, as assessed in our previous study^20^, could be extended to construct cell migration profiles that are distinguishable for different cell types. Hence, we investigated this idea by pooling together the *CN correlation* data for each cell type.

**Figure 2:**
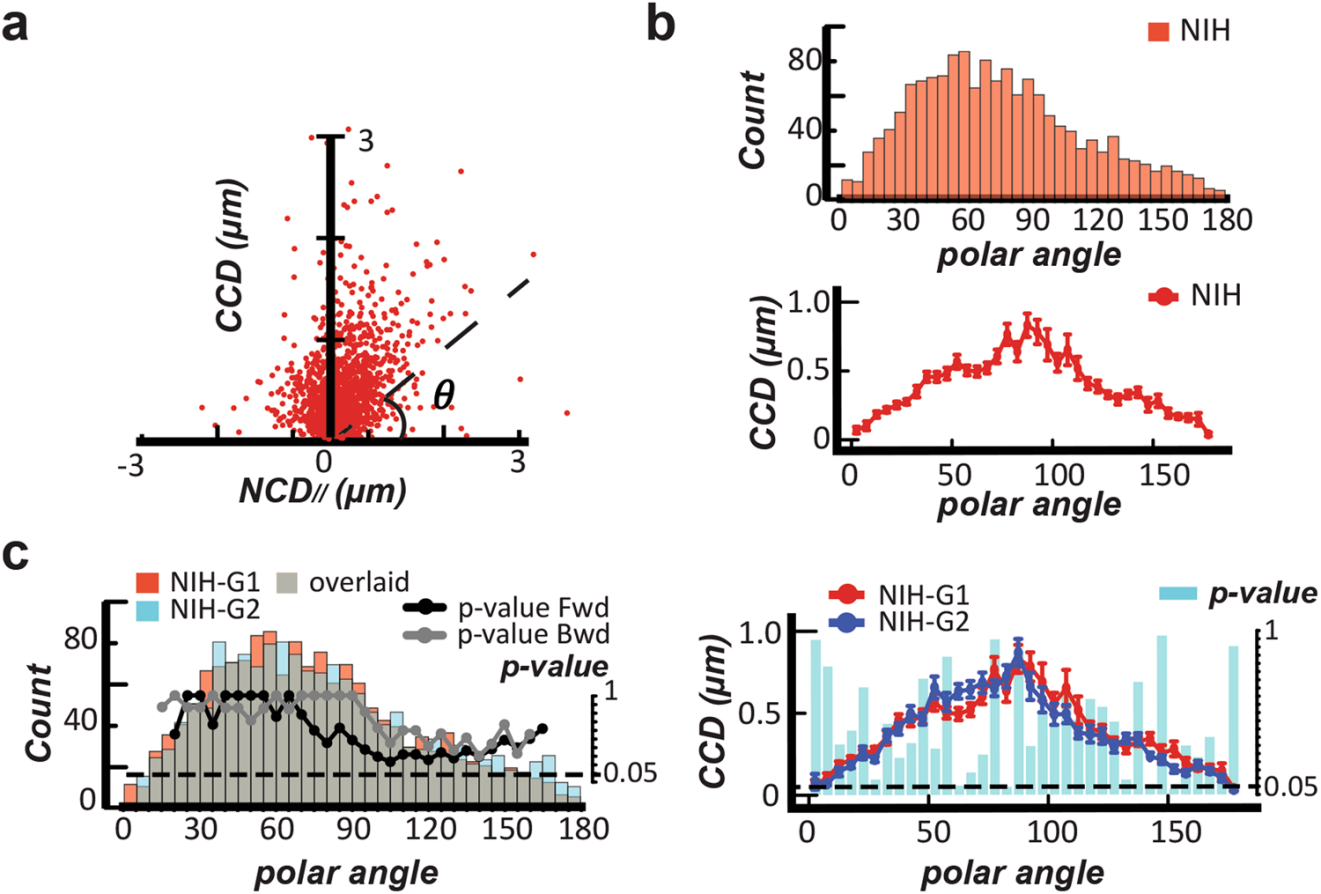
Distinct cell migration patterns can be distinguished by the CN correlation profiles. **(a)** A *CN correlation* profile (n = 1500) is constructed using a collection of 25 randomly selected one-hour movies of NIH fibroblasts at one-minute intervals. **(b)** A *CN correlation* profile can be separately presented by the histograms of angles (occurrence diagram, top) and the average *CCD* (<*CCD*>) over every 5°-polar angle bin, $\theta$ (*CCD* diagram, bottom). **(c)** Two occurrence diagrams and two *CCD* diagrams, constructed from independent *CN correlation* profiles of NIH 3T3 fibroblasts (NIH-G1 and NIH-G2), were compared using the Sign test (top) and the Lepage test (bottom). The resulting *p*-value is displayed by line and bar in the occurrence diagram and the *CCD* diagram, respectively. The right Y-axis represents the *p*-value, where the dotted line indicates the *p*-value = 0.05.

To construct a representative *CN correlation* profile for a specific cell type, a sufficient amount of randomly sampled *CN correlation* data is needed (Fig. 2a). Accordingly, we evaluated whether two *CN correlation* profiles extracted from different batches of the same cell type can be indistinguishable to illustrate the unique migration pattern of the probed cell type, and whether *CN correlation* profiles generated from different cell types are distinguishable. Two *CN correlation* profiles were constructed from NIH 3T3 fibroblasts (see supplementary information for other cell types). Each profile was generated from 25 randomly selected and non-overlapping one-hour cell movies at one-minute time intervals. Since the polar coordinates in the *CN* plot are connected to the biological meaning of the subcellular migratory activities, a useful and equivalent representation of (*NCD*_//_, *CCD*) pairs would be in terms of polar angles and the magnitude of *CCD values*. Hence, each *CN correlation* profile was summarized using histograms of angles (called occurrence diagram) and average-*CCD* (called *CCD* diagram), presented with 5°-polar angle bins (Fig. 2b). Then, the Sign test and the Lepage test were subsequently applied to compare the occurrence and the *CCD* diagrams from the two *CN correlation* profiles (Fig. 2c). These results revealed that the *CN correlation* profiles extracted from different batches of the same cell type are statistically indistinguishable for describing the specific cell migration pattern.

Consequently, the *CN correlation* profiles of eight cell types were individually constructed, including 1 epithelial cell: OSE-10; 3 fibroblasts: NIH 3T3, Swiss 3T3 and human foreskin; 3 adenocarcinoma cells: OVCAR-3, SKOV-3, and MDA-MB-231; and 1 osteosarcoma cell, U-2 OS. When these occurrence diagrams and *CCD* diagrams were paired for comparison using the Sign test and the Lepage test, respectively, the results clearly indicated significant differences between their profiles (see Supplementary Information). Hence, among all the cell types considered, each individual cell type possesses a unique *CN correlation* profile describing its cell migration pattern and momentary dynamics.

### The changes in a *CN Correlation* profile can reveal the effect of perturbation on cell migration

**Figure 3:**
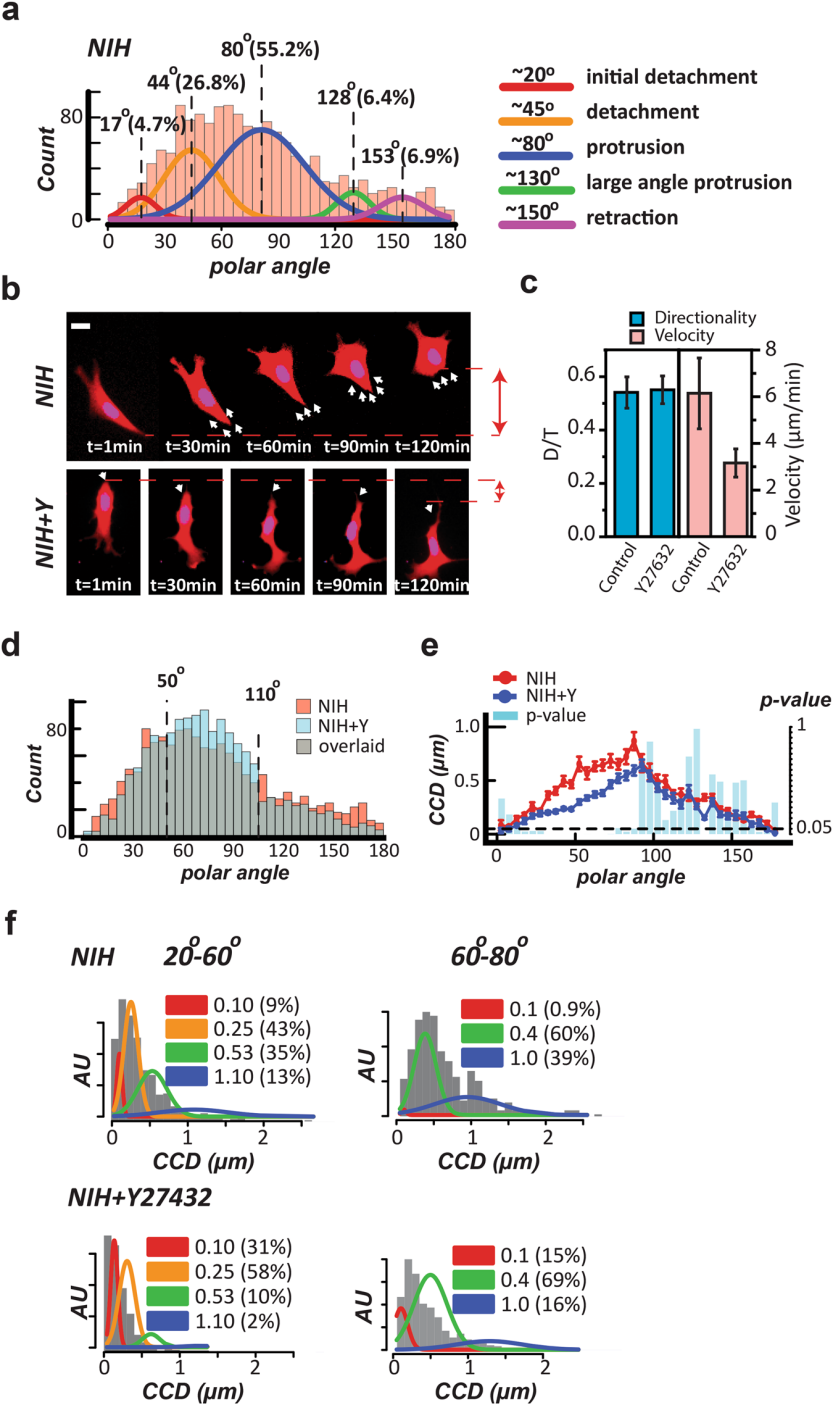
The *CN correlation* profiles can reflect changes in cell locomotion. **(a)** The univariate normal mixtures (*UNM*) analysis was applied to the occurrence diagram of NIH 3T3 fibroblasts (NIH) to profile the component normal distribution curves that describe the migration signature of the cell type. **(b)** Time-lapse images display the changes in the representative migration pattern between the normal NIH 3T3 fibroblasts and the same fibroblasts treated with Y27632. The white arrows indicate the directions of detachment. The red double-arrows indicate the total detached distance in two-hour intervals. Scale bar: 20 µm. **(c)** The directionality (D/T) and velocity of the normal (NIH) and the Y27632-treated (NIH + Y) cells were shown. Error bars indicate the standard error of the mean (SEM). **(d)** The comparison of the occurrence diagrams between the normal (NIH) and the Y27632-treated (NIH + Y) cells. **(e)** The comparison of the *CCD* diagrams between the normal and the Y27632-treated cells. The cyan bars show the *p*-value of the Lepage test between the two groups. Dotted line indicates where the *p*-value = 0.05 as indicated in the right Y-axis. **(f)** *Top:* The *UNM* analysis to the *CCD* diagram of NIH 3T3 fibroblasts yielded 4 normal distributions in the detachment zone (20°–60°) and 3 normal distributions in the protrusion zone (60°–80°). *Bottom:* The same analysis shows that Y27632 treatment drastically re-distributes the *CCD* values in both the detachment zone and the protrusion zone.

A *CN correlation* profile should be an unbiased representation of the overall contributing subcellular migratory activities of that cell type. Since each subcellular activity has a particular polar angle distribution, the component weights of each of these activities can be de-convoluted from the occurrence diagram using Gaussian mixture modeling. Hence, univariate normal mixtures (*UNM*) were applied to the occurrence diagram of the NIH 3T3 fibroblasts to automatically profile the normal distributions using the Aikake information criterion (AIC)^21^ (Fig. 3a). We called the fitted peak polar angles of these distributions the signature angles, which can be considered to be a property of the cell type. The result showed that the migration pattern of NIH 3T3 fibroblasts has 5 signature angles around 17°, 44°, 80°, 128°, and 153° polar angles. Based on the locations of the exampled individual subcellular activities in the *CN* plot, these normal densities can be considered to be sequentially corresponding with the initial detachments, detachments, protrusions, large-angle protrusions, and leading-edge retraction from the whole cell contraction, respectively.

Consequently, we applied a ROCK inhibitor, Y27632, to block the contractility of these cells and evaluated the changes in the cell motility signatures. The representative time-lapse images suggested that the Y27632-treated cell still had a persistent direction; however, its trailing-edge detachment rate was drastically decreased (Fig. 3b). We further quantitatively assessed the Y27632 effect using two widely-accepted conventional measurements, namely directionality and cell velocity^22^. The results indicated that Y27632 reduced ~45% of the NIH 3T3 fibroblasts’ velocity without affecting the directional persistence (Fig. 3c). For the analysis based on the *CN correlation* approach, the comparison of the occurrence diagrams between NIH 3T3 fibroblasts and their counterparts treated by Y27632 exhibited a slight decrease of the *CN correlation* occurrence in the polar angles below 50° and above 110° after drug treatment (Fig. 3d). This redistribution suggested that the cells have reduced detachment and large-angle protrusion events involving actomyosin contraction. Hence, the *CN correlation* profile is a sensitive descriptor for cell migration patterns.

Y27632 also can induce membrane ruffling^23^; hence, Y27632 should affect the <*CCD*> values as well. It was shown that the <*CCD*> values of NIH 3T3 cells decreased across the *CN* plot, with the most significant drop occurring in the 30°–80° polar angles (Lepage test: *p*-values < 0.001) (Fig. 3e). When the *UNM* analysis was applied to the *CCD* distributions in the detachment and protrusion polar angles to automatically profile the component weights in normal cells, we found that 4 normal distributions in the detachment zone and 3 in the protrusion zone. These signature distributions might be matched with immobile, asynchronous, synchronous, and abrupt detachment (from the cell contraction) mode for detachment events; and immobile, steady, and rapid protrusion mode for protrusion events (Fig. 3f). A comparison of the *CCD* distributions between the normal and the Y27632-treated cells suggested that ROCK inhibition significantly reduced the abrupt detachment mode and increased the immobile mode in the detachment events; meanwhile, it drastically reduced the immobile (stable anchorages) mode and rapid protrusion mode (requiring ROCK activity^24^), and increased the steady protrusion mode (membrane ruffling) in the protrusion events. The differences in the one-minute step sizes between the normal and the Y27632-treated cells correctly reflect the cellular effects of Y27632.

### The *CN correlation* profiles can characterize cell polarity of different cell types

**Figure 4:**
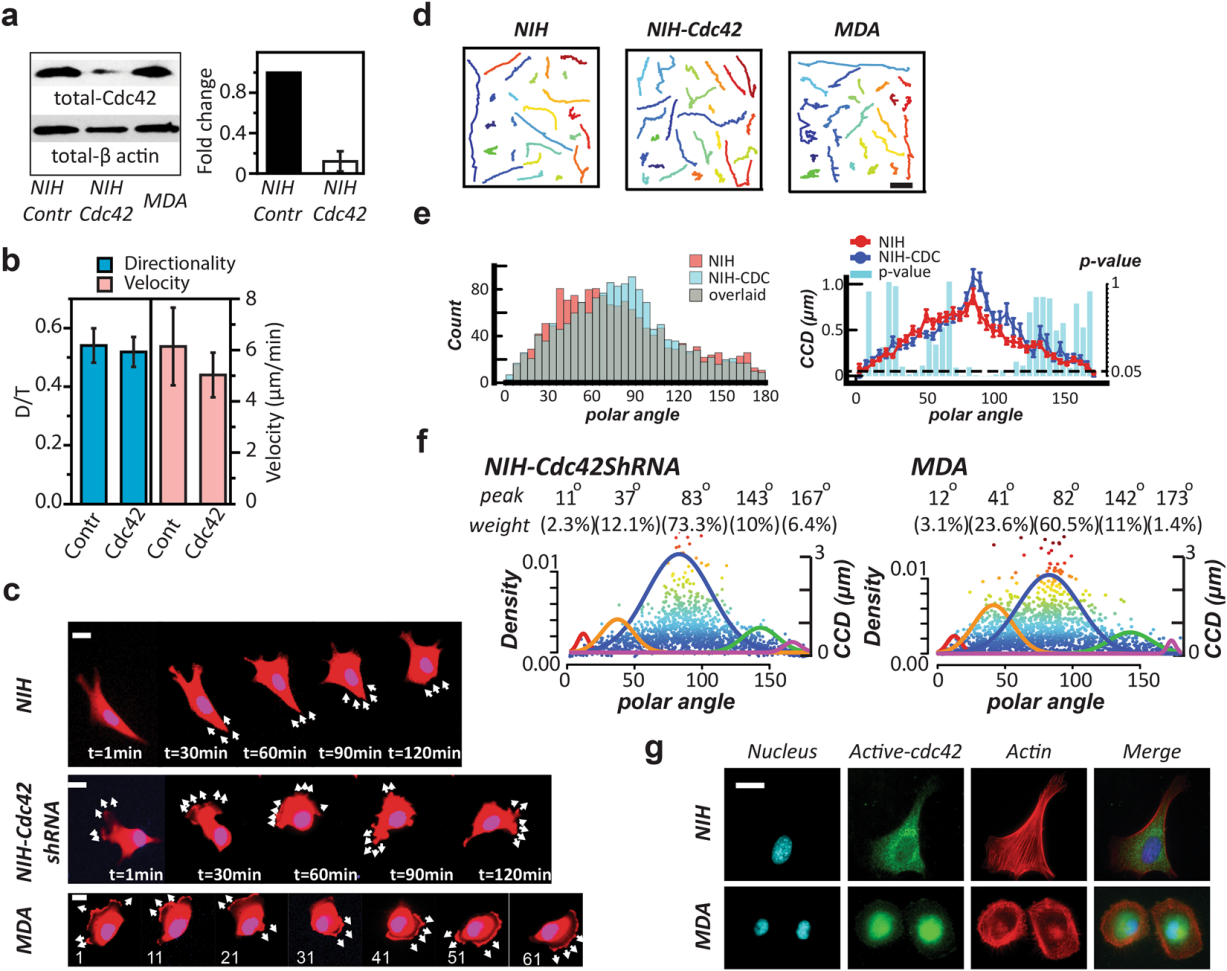
The *CN correlation* approach reflects the cell polarity changes. **(a)** Western blot exhibits the total amount of Cdc42 in NIH 3T3 fibroblast (NIH), NIH with Cdc42 knockdown, and MDA-MB-231 (MDA), from left to right, respectively (left). The q-PCR results show a significant decrease of fold change (normalized by GAPDH) in the Cdc42 mRNA amount in the NIH 3T3 fibroblasts after Cdc42 knockdown (right). Error bars represent the SEM. The full-length blots are presented in the Supplementary Figure S4. **(b)** The directionality (D/T) and velocity of the NIH control group and the NIH with Cdc42-knockdown group. **(c)** Time-lapse images display the migration pattern of a prototypical NIH 3T3 cells (NIH), MDA-MB-231 cells (MDA), and the NIH 3T3 cells with Cdc42-knockdown (NIH-Cdc42shRNA). Scale bar: 20 μm. **(d)** From the left to the right, the trajectories of NIH, MDA, and NIH-Cdc42shRNA, respectively. Scale bar: 20 μm. **(e)** The overlaid occurrence diagrams (left) and the overlaid <*CCD*> diagrams (right) of the NIH and the Cdc42-knockdown counterparts (NIH-CDC). In the <*CCD*> diagrams, each bar shows the Lepage test result of the *CCD* distributions in the designated 5° polar-angle zone. The dash line: *p*-value = 0.05. **(f)** The NIH with Cdc42 knockdown and MDA possess similar signature angles, analyzed by *UNM*. We use the *Signature Plot* to express the results, where the top two rows show the signature angles and the weighing percentages of the corresponding signature curve, the solid color normal distribution curves outline the individual profiling subcellular migratory activities with the left Y-axis labeling the occurrence rate, and the dots are the individual *CN correlation* data with the right Y-axis labeling the *CCD* value. **(g)** Fluorescent images display the distribution of active Cdc42 in the NIH and MDA cells. Scale bar: 20 μm.

Since Cdc42 is a protein that maintains cell polarity and NIH 3T3 fibroblasts usually have a relatively straight trajectory, we applied the Cdc42 knockdown to the fibroblasts to explore the migration pattern change. First, we confirmed the success of Cdc42 knockdown through western blot and q-PCR (Fig. 4a). Consequently, we compared the directionality and velocity between the normal fibroblasts and the counterparts with Cdc42 knockdown. The results indicate two indistinguishable groups (Fig. 4b**)**. However, from the time-lapse images (Fig. 4c) and trajectories (Fig. 4d), it was clearly seen that the Cdc42-knockdown cells have altered their migratory pattern from the normal NIH 3T3 fibroblasts. Interestingly, we found these Cdc42-knockdown cells have more similar features to those of the MDA-MB-231 cells in the morphology, migration patterns, and trajectories. Cdc42 knockdown seems to induce more frequent large-angle protrusions to the fibroblasts, resulting in less persistence in cell migration direction.

Among the 8 studied cells, MDA-MB-231 and NIH 3T3 cells are the most motile; however, MDA-MB-231 cells made turns more frequently than the NIH 3T3 fibroblasts. When we applied the *CN correlation* analysis, the changes in the migration patterns were elucidated clearly (Fig. 4e). We noticed that Cdc42 knockdown shifts the distribution of the *CN correlation* data of NIH 3T3 fibroblasts from the detachment zones (30°–60°) to the protrusion/side protrusion zones (70°–120°). These shifts were in agreement with the impression gained from the time-lapse images and the Cdc42 function, which is in maintaining the directional persistence during cell migration. In addition, the *CCD* diagrams also showed that Cdc42 knockdown resulted in *CCD* value increases in the protrusion and side protrusion zones. This increase implied that less regulations (*e.g*., filopodia formations) were imposed to the lamellipodia to define cell polarity due to the modulation of Cdc42 activity^25^.

Through the *CN correlation* Signature Plot, the NIH 3T3 fibroblasts with Cdc42 knockdown were compared to the NIH 3T3 fibroblasts and the MDA-MB-231 cells (Fig. 4e). We noticed that the Cdc42 knockdown shifted the signature angles of the fibroblasts to make them similar to those of the MDA-MB-231 cells. The NIH 3T3 fibroblasts had a much lower turning frequency than their CDC42 knockdown counterparts (signature angle/occurrence: 128°/6% *vs*. 143°/10%), which was approximately the same as the MDA-MB-231 cells (142°/11%).

In the cytoplasm, Cdc42 forms a complex with Par6 and Protein Kinase C-zeta (PKC-ζ) to regulate the reposition of microtubule organization center (MTOC)^18^. The western blot result suggested that MDA-MB-231 cells have a comparable expression level of Cdc42 to NIH3T3 fibroblasts (Fig. 4a). This seemingly generates an inconsistent perspective to the conclusion that Cdc42 knockdown causes the migration pattern of NIH 3T3 fibroblasts to be similar to that of MDA-MB-231 cells. Hence, we further applied immunofluorescence microscopy to examine the location of active Cdc42 in both cells. The micrographs showed that active Cdc42 only appears to the perinuclear region of the MDA-MB-231 cells (Fig. 4f). Cdc42 is known to regulate filopodia in the lamella region and activate the Par6/PKC-ζ complex to maintain cell polarity^17,18^. It has been shown that the amounts of Par6 and PKC-ζ are also abundant in the MDA-MB-231 cells; however, both Par6 and PKC-ζ fail to locate in the proximal region of the plasma membrane to maintain the cell polarity in MDA-MB-231 cells^2^. Taken together, these studies suggested that the active Cdc42 in the perinuclear region of the MDA-MB-231 cells has not triggered the Par6/PKC-ζ complexes for their normal function to maintain cell polarity. Here, the *CN correlation* approach serves as a direct metric to collectively reveal the integrated activity of cell polarity of the associated proteins.

### The *CN Correlation* profiles reveal cell motility through the component weights of active migration

Variants of random walk or persistence time models have been applied to single cell trajectories to estimate the rate of area coverage (*i.e*., diffusion coefficient). The persistence time provides a method for comparing the overall motility of different cell types. However, this type of approach cannot directly resolve the contributions of the individual subcellular migratory activities to overall motility. In contrast, the *CN correlation* approach assesses the migration process through the composition of subcellular migratory activities; hence, it can quantify the weights of subcellular activities for active migration. We thus utilized the *CN correlation* approach to summarize the overall motility of a cell type in terms of accumulated *NCD*_//_ from the active migrating region^20^. This estimation was called the cell migration potential index (*CMPI*) and thus serves as an empirical metric for comparing the motility among different cell types. Comparisons with existing single cell tracking approaches were also discussed in that previous work^20^.

**Figure 5:**
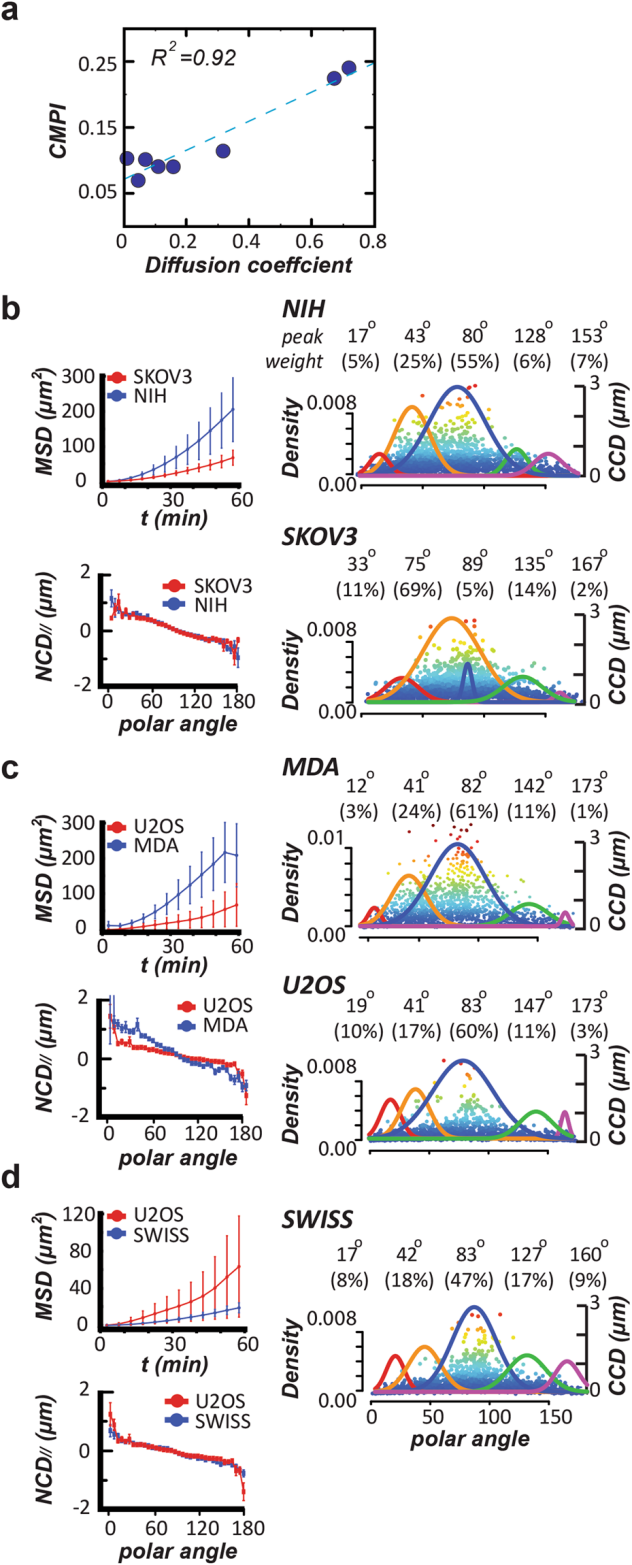
The *CN correlation* profile enables the estimation of cell motility. **(a)** Correlation between *CMPIs* and diffusion coefficients of persistence time for 8 different cell types. A blue dot represents a cell type, and the dashed line is the fitting curve. **(b)** *Left:* MSDs *vs*. time lags (top) and <*NCD*_//_> *vs*. every 5° polar-angle zone (bottom) of NIH3T3 (NIH) and SKOV-3 (SKOV) cells. *Right:* The *Signature Plots* of these two cells. **(c)** *Left:* MSDs *vs*. time lags (top) and <*NCD*_//_> *vs*. every 5° polar-angle zone (bottom) of MDA-MB-231 (MDA) and U-2 OS (U2OS) cells. *Right:* The *Signature Plots* of these two cells. **(d)** *Left:* MSDs *vs*. time lags (top) and <*NCD*_//_> *vs*. every 5° polar-angle zone (bottom) of U-2 OS and Swiss 3T3 (Swiss) cells. *Right:* The *Signature Plots* of Swiss 3T3 cells.

When the *CN correlation* profile was de-convoluted by the *UNM* analysis, we could isolate the *CN correlations* under active cell migration necessary to estimate *CMPI*. Even through the signature angle of the protrusion density curve indicates the relative contributions of different protrusion events, the *NCD*_//_ values associated with protrusion are usually small. The *CMPI* does not present an absolute displacement; rather, it is a relative quantity describing the effective migration events among different cell types. Hence, we used the *NCD*_//_ values of all the *CN correlation* data in the detachment zone to compute the *CMPI*. The detachment zone was determined by the polar angles in the *CN* plot smaller than the signature angle +2 standard deviations of the detachment curve (Fig. 5a). The results were fitted with the diffusion coefficients generated from persistence random walk model well (R^2^ = 0.92). Therefore, the *CN correlation* approach can be applied to examine the separate cell motilities caused by the varying migration patterns of different cell types.

The prototypical trajectories and mean-squared displacements (MSD) suggested that NIH 3T3 fibroblasts have significantly higher motility than the SKOV-3 cells. These two cells had almost identical average step sizes (<*NCD*_//_>) for each 5°-polar angle bin in their *CN correlation* profiles. In addition, approximately 85% of the *CN correlation* data of these two cells were accounted for within the first three component distributions. However, the *UNM* analyses showed that the NIH 3T3 fibroblasts had significant lower signature angles in the subcellular migratory activities than SKOV-3 cells (Fig. 5b). Hence, the NIH 3T3 fibroblasts should have more active migration and a higher motility with respect to the SKOV-3 cells, which instead have more sampling activities.

When the comparison was made between MDA-MB-231 and U-2 OS cells, the prototypical trajectories and the MSDs indicated MDA-MB-231 cells are more motile. The Signature Plots showed that ~ 88% of the *CN correlations* of both cell types were accounted for within the first three component distributions and all of the five signature angles were similar (Fig. 5c). However, in the first half of the *NCD*_//_ diagram, the MDA-MB-231 cells had significantly higher <*NCD*_//_> values contributing to motility, thereby having higher overall motility than U-2 OS cells.

U-2 OS cells have higher motility than Swiss 3T3 cells do, supported by the comparison of their MSDs and diffusion coefficients^20^. These two cells not only have nearly identical <*NCD*_//_> step sizes over the whole range of polar angles but also shared similar signature angles for the first three subcellular migratory activities (Fig. 5d). However, the occurrence rate of the protrusion events was 60% for the U-2 OS cells in contrast to 47% for the Swiss 3T3 fibroblasts. This greater occurrence rate suggested that the U-2 OS cells developed more stable protrusion features (lamellipodia) than Swiss 3T3 cells. The stable lamellipodia resulted in less effective leading-edge retraction in U-2 OS cells. According our prior analysis, during the formation of large-angle side protrusion, an effective leading edge retraction would prevent a significant nucleus forward motion; vice versa, an obstructive leading edge retraction (*i.e*., stable protrusion feature) would promote more significant nucleus forward motion. This could explain why the U-2 OS cells have a much larger signature angle (147°) for the large angle protrusion curve (smaller *CCD* and greater *NCD*_//_ values) than the Swiss 3T3 fibroblasts do (127°).

In summary, our proposed *CN correlation* approach provides a clear link between subcellular migratory activities and cell motility. Cell motility is positively correlated with the occurrence rates of the highly polarized subcellular migratory activities, in which the polar angles of the corresponding *CN correlations* mainly locate <80°-polar angle in the *CN* plot. In addition, the cell motility also significantly depends on the magnitude of the nucleus step sizes in the active migration. Together, the *CN correlation* profile can serve as a valuable platform for documenting cell migration patterns and elucidating the effects of perturbations on distinct subcellular migratory activities, motility, and cell polarity state.

## Discussion

This study has the overarching goal of building a multi-scale integration method to monitor cell dynamic phenotypes, such as cell migration. This approach utilizes the relative motions between individual cell centroid displacements and the concomitant nuclear centroid displacements (the *CN correlation* data) to quantitatively characterize the commonly known subcellular dynamic activities in probed cells. On the *subcellular scale*, each *CN correlation* datum is based on one-minute centroid displacements to express the momentary cell and nuclear motions. This information reveals the evolution of individual subcellular dynamic activities in the *CCD-N*CD_//_ coordinate system, thus promoting our understanding of the step-by-step transient mechanical mechanisms and elucidating the spatiotemporal relationships of the involved proteins in the associated signaling pathways.

We note that in the previous study^20^, we reported the *CMPI* as a quantitative parameter. Herein, we showed that the cell migration pattern proposed can also be used in a similar way as the *CMPI*, but is superior because it is more comprehensive. For example, the cell migration pattern (unlike the *CMPI*) reveals the percentage of sub-cellular phenotypic events that contribute to cell migration. These new developments can pave the way for connecting these phenotypic events to molecular events in the future.

On the *cellular scale*, we characterize the comprehensive cell dynamic pattern of the probed cell type by the extracted *CN correlation* profile from representative samples of 25 randomly selected one-hour movies. *CN correlation* profiles were shown to be reproducible between batches of the same cell types, yet significantly different among a variety of distinct cell types. Hence, this study provided empirical evidence showing that a cell type possesses a consistent and unique dynamic pattern at a defined culture condition. The *CN correlation* profile is a powerful platform to present the cellular dynamic signature, which distinguishes unique cell dynamic patterns along with the impacts of biochemical and biophysical cues on events such as migration.

When the *CN correlation* approach is used to for the *intercellular scale* (*i.e*., bridging the subcellular and cellular scales), we demonstrated that a *CN correlation* profile can be analyzed to elucidate the weighing contributions of the subcellular migratory activities, either in a single cell migration event or in the cell migration pattern of a given cell type. Once the signature of a *CN correlation* profile is de-convoluted statistically through the univariate normal mixtures (*UNM*), the component distributions of the underlying subcellular activities to the cell migration process (or pattern) can be recognized. This capacity allows us to effectively extract the time duration of directional persistent motions during cell migration to estimate cell motility and to trace the molecular causes for a motility change.

Since the *CN correlation* approach carries the capacity to link the multi-scaled information together, the tight combination of this approach and other available cell dynamic knowledge can greatly promote our comprehensive understanding to cell phenotypes in which cell dynamics is a concern. When a stimulus (such as a drug and a protein mutation/knockdown) is present to alter the cell dynamic phenotype, it distributes the effects through individual subcellular activities that are separately governed by the Rho GTPases, RhoA, Rac1, and Cdc42, where the signaling of these pathways respectively for cell contracting, expanding, and direction-persisting. Hence, the *CN correlation* metrics can clearly elucidate the re-distribution of the individual pathways to decipher the complexity of a signaling event that has interactions with the Rho GTPases to change the cell dynamics.

In this study, we demonstrated this capacity of *CN correlation* using a ROCK inhibitor and Cdc42 knockdown. With respect to the conventional cell motility methods, the *CN correlation* approach can help provide clearer insights in a quantitative context. While NIH 3T3 fibroblast with Cdc42 knockdown and MDA-MB-231 cells exhibit similarities in maintaining polarity, they have very different molecular contents. First, they are very different cells even in the genotypes (*i.e*., mouse *vs*. human). In addition, MDA-MB-231 cells have high expression levels of Cdc42, Par6, and PKC-ζ, while the Cdc42 is knocked down in the NIH 3T3 fibroblasts. The *CN correlation* metric can affirmatively provide a solid assessment of the cell migration pattern, including cell polarity, toward establishing the detailed molecular mechanisms.

It is known that the predominant dynamic activity of epithelial cells is protrusions and the migration of fibroblasts (the mesenchymal-like cells) involves trailing-edge detachments. From the *CN correlation* concept, a detachment event will have *NCD*_//_ > *CCD*. Hence, a good portion of *CN correlation* data should distribute within the polar angles between 30° and 45° during mesenchymal migration (Table S1). The *CN correlation* approach clearly exhibits these weight differences between cells with epithelial phenotypes (OSE10 and OVCAR-3 cells) and mesenchymal-like phenotypes (human foreskin, Swiss 3T3 and NIH 3T3 fibroblasts, and SKOV-3, MDA-MB-231 and U-2 OS cells). All the mesenchymal-like cell lines possess a signature angle within the detachment region. Meanwhile, no signature angle has been found within this region for probed epithelial cells. This leads to a question that remains for further investigation: should a signature angle appearing in the detachment region (*i.e*., 30°–45°) be considered as a criterion to distinguish cells between epithelial and mesenchymal types of migration? In addition, even though the *CN correlation* has provided a measurable outcome for the cellular/subcellular activities, the detailed molecular mechanisms that cause the differences of the outcomes require comprehensive molecular information of the underlying pathways, as the example in the CDC42 study here shows.

The ultimate goal of integrated biology focuses on organizing all involved molecular mechanisms to explain the cellular processes in the higher levels. While the spatiotemporal dynamics of the subcellular activities at the molecular levels has been gradually revealed using novel techniques such as fluorescent speckle microscopy^4^, the spatiotemporal dynamics among the subcellular activities leading to a specific cellular phenotype has been the focus of this study. Currently, a successful effort of integrated biology requires precise molecular information from genomic and proteomic methods as well as correct mathematical models to describe the cellular process. The lack of spatiotemporal information in the genomic and proteomic measurements hinders the effective promotion of such efforts. Furthermore, the complicated signaling crosstalk also greatly obstructs the building of precise mathematical models to facilitate cellular behavior prediction. For drug screening, cancer diagnosis, and the study of certain molecular mechanisms that are involved in cell dynamic changes, the *CN correlation* approach can provide a promising and innovative cell migratory signature with straightforward biological meaning to overcome these challenges.

## Methods

### Cell cultures and substrate

All media were from Mediatech (Manassas, VA). NIH 3T3, Swiss 3T3, human foreskin fibroblasts and MDA-MB-231 cells (all from ATCC, Manassas, VA) were maintained in DMEM media with 10% fetal bovine serum (FBS) and 1% L-glutamine. OSE-10, OVCAR-3, and SKOV-3 were gifts from Dr. Ie-Ming Shih (JHMI) and were cultured in RPMI 1640 medium, containing 10% FBS, 1% L-glutamine, and 1% penicillin-streptomycin. U-2 OS cells (ATCC) were cultured in McCoy’s 5 A medium, containing 10% FBS and 1% L-glutamine. These cells were kept in a humidified incubator at 37 °C and 10% CO_2_.

### Cell sample labeling, treatment and drug application

For RFP labeling, pRFP-R-CS plasmids (Origene, Rockville, MD) were introduced to culture cells using transfection by Lipofectamine 2000 reagent (Invitrogen, Carlsbad, CA). Hoechst 33342 (Sigma-Aldrich, St. Louis, MO) was applied to cell culture ten minutes prior to image acquisition. For Cdc42 knockdown, the Cdc42shRNA plasmids (Origene) were co-transfected with the pRFP-R-CS plasmids. For Y27632 (Millipore, Billerica, MA) treatment, 20-µM was applied 30 min prior image acquisition.

### Quantitative-PCR

The RNeasy Mini Kit (Qiagen, Valencia, CA) was applied to isolate the total mRNA from cell samples. The iScript Advanced cDNA Synthesis Kit (Bio-Rad, Hercules, CA) was used to reverse-transcribe the isolated mRNA to cDNA. Consequently, the quantities of the target cDNA were determined by quantitative PCR (qPCR) using CFX connect system (Bio-Rad) with SYBR supermix (Bio-Rad). GAPDH were assessed as the standard in different samples. Intron-spanning primers for Cdc42 (Genbank accession number: U37720.1) and GAPDH (GU214026.1) were designed using Primer3 and synthesized by Integrated DNA Technologies (Skokie, IL) as follows:

Cdc42 sense: 5′-CTGGGGCATCTTCGTGTCTT-3′;

Cdc42 antisense: 5′-AACCCCATACACACCCCAAA-3′;

GAPDH sense: 5′-TCTCTGCTCCTCCCTGTTCC-3′;

GAPDH antisense: 5′-GTTCACACCGACCTTCACCA-3′.

Nine measurements (3 samples, each repeated 3 times) were performed and the results were determined by the comparative Ct method.

### Western blot

Cells were seeded at 5 × 10^4^ cells/ml and cultured overnight before harvest. Each protein extract was obtained from a cell sample, scratched and harvested using cell lysis buffer (Cytoskeleton Inc., Denver, CO) on ice, and denatured using 5× Laemmle sample buffer. The samples were separated by 12% SDS-PAGE, transferred onto a PVDF membrane, and probed with primary antibodies against Cdc42 (BD biosciences, Bedford, MA) and Beta-actin (Abcam, San Francisco, CA). After horseradish peroxidase-conjugated secondary antibody was against the primary antibody, the image was developed using standard protocol provided by the vender (Thermo Fisher Scientific, Waltham, MA).

### Cell immunofluorescence staining

Cells were seeded on fibronectin (20 µg/ml)-coating, glass-bottom dishes and cultured overnight. Samples were fixed by 4% paraformaldehyde in PBS for 20 min in room temperature, and permeabilized by 0.5% Triton X-100 in PBS for 5 min. After samples were blocked with 5% BSA in PBS for 1 hour, samples were stained with primary antibody active-Cdc42-GTP (New East Biosciences; 1:100 dilution) for 3 hours, and then subjected to Alexa Fluor 488-conjugated anti-mouse IgG (Invitrogen; 1:1000 dilution) for 1 hour at room temperature. Actin and nuclear were stained by Alexa Fluor 568-phalloidin (Sigma-Aldrich; 1:40 dilution) and Hoechst 33342 (Sigma-Aldrich; 1:100 dilution).

### Image acquisition and analysis

A TE-2000 Image acquisition system (Nikon, Melville, NY), equipped with Cascade:1 K CCD camera (Roper Scientific, Tucson, AZ), was used to acquire images. For the movies, the setting was 100-ms exposure time and 3 × 3 binning and acquired by Images were acquired using a 20× objective lens. The setting was 100-ms exposure time and 3 × 3 binning. The static images were acquired using a 60× oil objective lens. The cell samples were cultivated on the fibronectin-coating glass bottom dishes. When analysis, the geometric centroids of the cells were determined using a published method^26^.

### Directionality and velocity assessments

The directionality (D/T) was calculated by the ratio of distance between the starting and ending points during the observation time (D) to the sum of each displacement between the time lags (T). The observation time was set to every 10 min by overlapping time lags. The cell velocity was extracted by one-hour durations with overlapping time lags.

### Univariate Normal mixture analysis

The occurrence diagram and *CCD* diagram of the *CN correlation* profile were de-convoluted into normal distribution curves by univariate normal mixtures (UNM) fitting The function *normalmixEM* in the R package *mixtools* implements this algorithm^27^ (*R* Foundation for Statistical Computing, Vienna, Austria. URL: https://www.R-project.org/).

## Electronic supplementary material

Movie S1

Movie S2

Movie S3

Movie S4

Movie S5

Supplementary Information
