# Supplementary material for: Integrating transient cellular and nuclear motions to comprehensively describe cell migration patterns
Source: Sci Rep. 2018 Jan 24;8:1488. doi: 10.1038/s41598-018-19885-y (PMC5784082; doi:10.1038/s41598-018-19885-y)
Supplement: Supplementary file 6 — Supplementary Information [file 41598_2018_19885_MOESM6_ESM.pdf]

**Integrating transient cellular and nuclear motions to comprehensively describe cell migration patterns**

Lan Tian[1], Shen-Hsiu Hung[1], Xudong Su[1], Samuel W.K. Wong[2] and Yiider Tseng[1,3,4,5,†]
[1]Department of Chemical Engineering, [2]Department of Statistics, [3]J. Crayton Pruitt Family Department of Biomedical Engineering, [4]Institute for Cell & Tissue Science and Engineering, University of Florida, and [5]National Cancer Institute-Physical Science Oncology Center, Gainesville, FL 32611, USA.

[†]Correspondence should be addressed to:
Yiider Tseng, Ph.D.
PO Box 116005
University of Florida
Gainesville, FL 32611-6005
Tel: (352) 392-0862
Fax: (352) 392-9513
Email: ytseng@che.ufl.edu

## SUPPLEMENTARY DOCUMENTS

### SI Experiment Protocol

***Coating procedure***

The glass-bottom dishes (World Precision Instruments, Sarasota, FL) were subjected to a fibronectin-coating procedure for cell experiments. The dishes were treated with 1 M hydrochloric acid at 65°C for 4 hours. Consequently, phosphate buffered saline (PBS) was applied to rinse and remove the acid solution. Afterward, 0.01% poly-L-lysine (Sigma-Aldrich, St. Louis, MO) was loaded on the glass surface for 20 min before PBS-rinse again. Finally, 20-µg/ml fibronectin (BD Biosciences, San Jose, CA) was loaded to coat the surface for 1 hour at room temperature.

***Live cell image quantification***

A TE-2000 image acquisition system (Nikon, Melville, NY), equipped with a Cascade:1K CCD camera (Roper Scientific, Tucson, AZ) and a X-Cite 120PC fluorescent light source (EXFO, Ontario, Canada), was used for image acquisition. The microscopy was set at 100-ms exposure time, 3×3 binning, and 25% light source power. During experiments, an on-stage incubator with a $CO_2$ supplementary system (In Vivo Scientific, St. Louis, MO) was operated to maintain the $CO_2$ supply at 10% and the temperature at 37 °C. Cell and nucleus centroids were determined from the sequentially acquired images on each image frame. Afterward, the PDS-1000/He system (Bio-Rad Inc., Hercules, CA) was used to ballistically embed 100-nm fluorescent carboxylated polystyrene fluorophores (Invitrogen, Carlsbad, CA) onto the culture dishes. These beads were used as the fixed reference points to align the sequential microscopic image frames to minimize the error generated due to stage movements[1,2].

***Persistent random walk model analysis***

We used 10-hour nuclear centroid trajectories to obtain the overlapping mean squared displacement (*MSD*) of a probed cell type,

$$MSD(\tau) = \langle [x(t+\tau) - x(t)]^2 + [y(t+\tau) - y(t)]^2 \rangle,$$

where τ indicates the time interval, and we use the notation ⟨… ⟩ to indicate mean over time. The diffusion coefficient of each individual cell type, *D*, was calculated as $D = S^2P/2$, Where *S* and *P* represent the cell speed and the persistence time, respectively, obtained from fitting the *MSD* to the persistent random walk (*PRW*) model[3]:

$$MSD = 2S^2P\left[t - P\left(1 - e^{-t/P}\right)\right].$$

**SI Text**

**Cell turning collectively resembles the *CN correlation* distributions of assembled subcellular events**

A cell turning event is composed of several subcellular migratory events. As a result, the overall distribution of *CN correlations* of a cell turning event would be expected to be a composition of the distributions from the assembled subcellular migratory activities. Yet, from an example movie we found that a whole-cell contraction process could distribute some *CN correlation* data to the polar angle zone greater than 130° (**Fig. S1**).

The example movie showed that the whole cell abruptly contracted at the beginning of the turning event ($1^{st}$ - $8^{th}$ minute). This contraction led the momentary leading-edge retraction and generated a sequence of *CCD* in a direction opposite to previous movement. Meanwhile, the nucleus still performed a forward motion toward the leading edge, suggesting that a contractile force continuously existed between the leading edge and the nucleus. Hence, the coupled $NCD_{//}$ were opposite to the *CCD* direction and the corresponding *CN correlations* were distributed in the polar angle zone greater than 130°. In the case a tensile force did exist between the nucleus and the original leading edge, the leading edge and the nucleus underwent a so-called "tug-of-war" process. Hence, the tensile force would be released quickly when one of them withdraws significantly. Therefore, a greater *CCD* would lead to a smaller $NCD_{//}$ and vice versa.

Afterwards, the cell mainly performed sampling ($9^{th}$ – $25^{th}$ minute) and gave rise to *CN correlations* within the 60°-120° polar angle zone. Since the cell had already been released from the previous polarity, the previously elongated nucleus also exhibited a notable relaxation to become rounded. During the period, the cell eventually developed a more significant protrusion as the new leading edge. Hence, the nucleus rotated accordingly and the cell started protruding along the new direction ($26^{th}$ – $43^{rd}$ minute). In this period, the *CN correlation* data were distributed in a similar manner as protrusion under active migration cases. Following, the cell performed regular detachment under active motion ($44^{th}$ – $60^{th}$ minute).

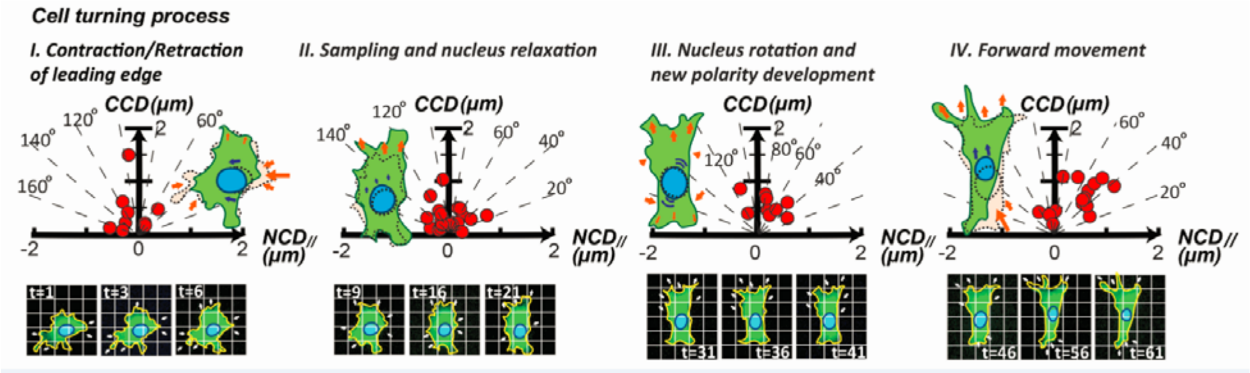


**Figure S1. In a cell turning event the *CN correlations* distribution collectively resembles those of the assembled subcellular events.** A NIH 3T3 fibroblast turning event (Movie S5) consists of 4 sequential and independent subcellular migratory events: leading edge retraction, cell sampling with notable nucleus shape adjustment, nucleus rotation and new polarized direction development, and active leading-edge protrusion (from left to right). The *CN correlation* distribution of the corresponding event (red dots) is displayed in the *CN* plot. The schematic diagram of each assembled subcellular migratory activity is also depicted in the associated panel. The overlaid time-lapse frames of fluorescence cell (green) and nucleus (blue) are displayed in grim graphs (bottom row).

**The *CN correlation* profile of a cell type is consistent and unique**

To further prove that the *CN correlation* profile of a cell type is representative and unique, we evaluated two *CN correlation* profiles extracted from different batches of the same cell type. Besides fibroblasts (NIH 3T3 fibroblasts) (in main text **Fig. 2c**), different cell types, such as osteosarcoma cells (U-2 OS cells) and adenocarcinoma cells (SKOV-3 cells), all constructed indistinguishable *CN correlation* profile (**Fig. S2a**). In

addition, we also tested whether the *CN correlation* profiles among different cell types are distinguishable. Eight cell types were cross-compared, including 1 epithelial cell (OSE-10), 3 fibroblasts (NIH 3T3, Swiss 3T3 and Human Foreskin (HF) fibroblasts), 3 adenocarcinoma cells (OVCAR-3, SKOV-3, and MDA-MB-231 cells), and 1 osteosarcoma cell (U-2 OS cells). The *CN correlation* profiles of these 8 cell types were individually constructed using 25 one-hour cell movies recoded at one-minute time intervals. The occurrence diagrams and *CCD* diagrams were paired for comparison using the Sign test and the Lepage test, respectively. Since the comparisons of the other cell types have been shown in the later section of the main text, here we only showed the comparison between two cell types, U-2 OS cells and Swiss 3T3 fibroblasts, which have the most similar *CN correlation* profiles. The results clearly elucidated the significant difference between their profiles (**Fig. S2b**). Hence, among all the cell types considered, each individual cell type possesses a unique *CN correlation* profile describing its cell migration pattern and momentary dynamics.

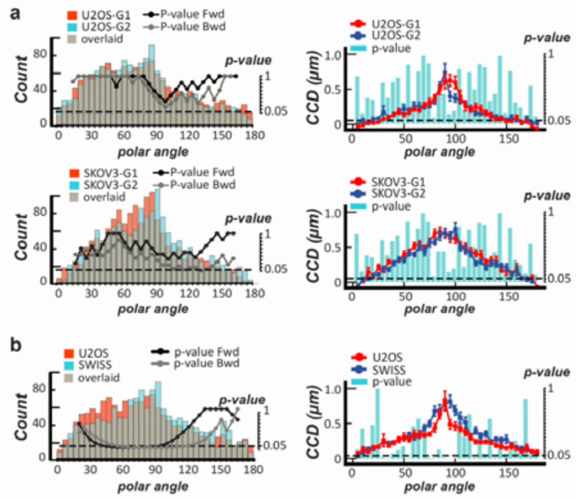


**Figure S2. The CN correlation profiles can distinguish the cell migration patterns of different cell types. (a)** Two individual *CN correlation* profiles constructed from the same cell types exhibit non-distinguishable patterns. Top: U-2 OS; bottom: SKOV-3. **(b)** The *CN correlation* profiles from two cell types with similar migrating behaviors, U-2 OS (red) and Swiss 3T3 (blue), are significantly different. Each profile consists of 25 randomly selected, one-hour, single-cell movies at one-minute intervals. The occurrence histogram of *CN* polar angle (left) and *CCD* diagrams (right) of each cell types were collected by five-degree and compared using the Sign test and the Lepage test, respectively.

**Hoechst 33342 staining will not affect the assessment of subcellular activities**

In the *CN correlation* approach, the imaging acquisition takes one-hour duration; the Hoechst 33342 stain was only applied to the sample 10 minutes prior to the acquisition. To explore whether the application of the Hoechst 33342 affects the imaging acquisition and the reliability of the *CN correlation* approach, we compared the subcellular morphology changes per minute by the axis ratio, the max length, the eccentricity and the circularity in individual cells within different one-hour durations using the two-sample-T-test under the following conditions: without staining, the first hour after staining, and the second hour after staining (**Table S2**). In addition, we also evaluated the histogram of cell dynamic activities during the first hour and the second hour after the Hoechst stain was applied (**Fig. S3**). These results demonstrated that no noticeable subcellular dynamic differences among the control and after the sample was subjected to the Hoechst stain.

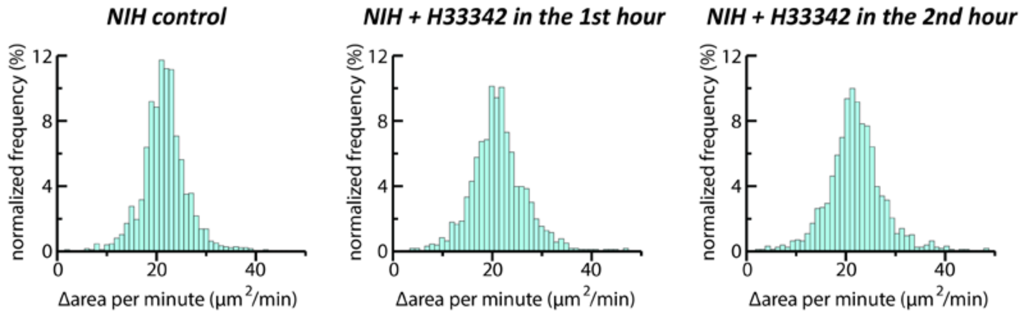


**Figure S3. The subcellular dynamic activities in the short-time intervals are consistent between the control cell samples and**

**the Hoechst 33342-applied cell samples for the first 2 hours.** The histograms of normalized changes of area per minute among the control cells, the Hoechst 33342-applied cells in the first hour, and the Hoechst 33342-applied cells in the second hour. The morphology changes were collected and analyzed through the boundary of GFP-transfected NIH 3T3 fibroblasts (NIH). Data was collected by at least 10 individual cells in one-hour observation times. Left to right: NIH control, the first hour of H33342 staining, and the second hour of Hoechst 33342 staining.

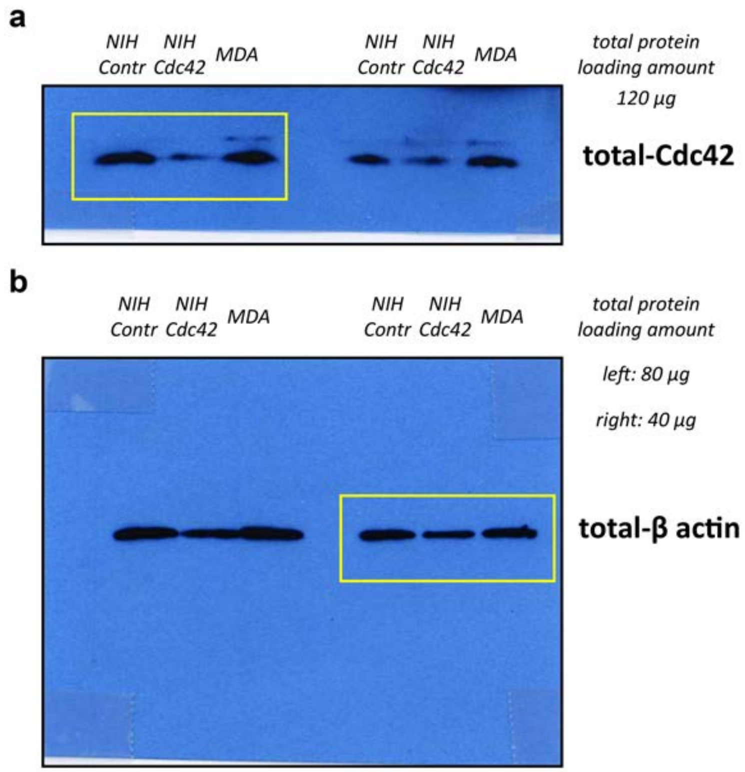


**Figure S4. Full-length blots of NIH 3T3 fibroblasts, their Cdc42-knockdown counterpart, and MDA-MB-231 cells. (a)** Two repeats of the total-Cdc42 amount. **(b)** Two repeats of the total-β actin amount. Yellow-boxes marks the blots represented in Fig. 4.

## Supplementary Table

| | HFF | | | NIH | | | SWISS | | | OSE | | |
|---|---|---|---|---|---|---|---|---|---|---|---|---|
| | weight | θ ° | height (μm) | weight | θ ° | height (μm) | weight | θ ° | height (μm) | weight | θ ° | height (μm) |
| c1 | 3% | 9 | 0.07 | 5% | 17 | 0.18 | 8% | 17 | 0.11 | 7% | 21 | 0.14 |
| c2 | 22% | 35 | 0.17 | 25% | 43 | 0.46 | 18% | 42 | 0.24 | 51% | 63 | 0.71 |
| c3 | 59% | 77 | 0.23 | 55% | 80 | 0.48 | 47% | 83 | 0.71 | 8% | 91 | 1.05 |
| c4 | 12% | 140 | 0.15 | 6% | 128 | 0.36 | 17% | 127 | 0.32 | 27% | 110 | 0.64 |
| c5 | 4% | 166 | 0.05 | 7% | 153 | 0.17 | 9% | 160 | 0.09 | 7% | 155 | 0.25 |
| | SKOV3 | | | MDA | | | U2OS | | | OVCAR | | |
| | weight | θ ° | height (μm) | weight | θ ° | height (μm) | weight | θ ° | height (μm) | weight | θ ° | height (μm) |
| c1 | 11% | 33 | 0.35 | 3% | 12 | 0.20 | 10% | 19 | 0.14 | 5% | 19 | 0.11 |
| c2 | 69% | 75 | 0.58 | 24% | 41 | 0.54 | 17% | 41 | 0.23 | 26% | 49 | 0.24 |
| c3 | 5% | 89 | 0.50 | 61% | 83 | 1.32 | 60% | 83 | 0.57 | 55% | 95 | 0.34 |
| c4 | 14% | 135 | 0.33 | 11% | 143 | 0.36 | 11% | 147 | 0.12 | 10% | 139 | 0.15 |
| c5 | 2% | 167 | 0.12 | 1% | 173 | 0.14 | 3% | 173 | 0.05 | 4% | 164 | 0.10 |

**Table S1.** The results of the univariate normal mixtures (*UNM*) analyses using *normalmixEM* procedure in the occurrence diagrams of 8 different cell types. Abbreviations for cell types: MDA-MB-231 (MDA), NIH 3T3 (NIH), SKOV-3 (SKOV), OVCAR-3 (OVCAR), OSE10 (OSE), U-2 OS (U2OS), SWISS 3T3 (SWISS) and Human Foreskin Fibroblasts (HFF).

| | ***T*-test: area changes** | | | ***T*-test: eccentricity changes** | | |
|---|---|---|---|---|---|---|
| | Control | 1st hour | 2nd hour | Control | 1st hour | 2nd hour |
| Control | - | 0.491 | 0.805 | - | 0.444 | 0.394 |
| 1st hour | | - | 0.342 | | - | 0.871 |
| 2nd hour | | | - | | | - |

| | ***T*-test: axis ratio changes** | | | ***T*-test: circularity** | | | ***T*-test: max length changes** | | |
|---|---|---|---|---|---|---|---|---|---|
| | Control | 1st hour | 2nd hour | Control | 1st hour | 2nd hour | Control | 1st hour | 2nd hour |
| Control | - | 0.577 | 0.617 | - | 0.451 | 0.619 | - | 0.293 | 0.510 |
| 1st hour | | - | 0.919 | | - | 0.768 | | - | 0.599 |
| 2nd hour | | | - | | | - | | | - |

**Table S2.** The *p*-value of two-sample-T-test of the subcellular activities between NIH 3T3 fibroblasts, the first and second hour after Hoechst 33342 was applied to the cells.

## Supplementary movies

**Movie S1-S5.** Each video file displays a typical cell migration mode at 1-min intervals over a 20-minute period, including (**Movie S1**) detachment, (**Movie S2**) protrusion, (**Movie S3**) sampling, (**Movie S4**) side protrusion, and (**Movie S5**) large angle turning. The file was an overlay of two simultaneous movies of the same cell: one documented the red fluorescence protein (RFP)-labeled cell and the other documented its coupled Hoechst 33342-labeled nucleus.

**Supplementary References**


1 Wu, P. H., Arce, S. H., Burney, P. R. & Tseng, Y. A novel approach to high accuracy of video-based microrheology. *Biophys J* **96**, 5103-5111, doi:10.1016/j.bpj.2009.03.029 (2009).

2 Wu, P. H., Nelson, N. & Tseng, Y. A general method for improving spatial resolution by optimization of electron multiplication in CCD imaging. *Opt Express* **18**, 5199-5212 (2010).

3 Stokes, C. L., Lauffenburger, D. A. & Williams, S. K. Migration of individual microvessel endothelial cells: stochastic model and parameter measurement. *J Cell Sci* **99 ( Pt 2)**, 419-430 (1991).
